# Variation in general practitioners’ follow-up of depressed patients starting antidepressant medication: a register-based cohort study

**DOI:** 10.1093/fampra/cmae063

**Published:** 2024-11-20

**Authors:** Anneli B Hansen, Øystein Hetlevik, Valborg Baste, Inger Haukenes, Tone Smith-Sivertsen, Sabine Ruths

**Affiliations:** Research Unit for General Practice, NORCE Norwegian Research Centre, Årstadveien 17, N-5009 Bergen, Norway; Department of Global Public Health and Primary Care, University of Bergen, Årstadveien 17, N-5009 Bergen, Norway; Department of Global Public Health and Primary Care, University of Bergen, Årstadveien 17, N-5009 Bergen, Norway; National Centre for Emergency Primary Health Care, NORCE Norwegian Research Centre, Årstadveien 17, N-5009 Bergen, Norway; Research Unit for General Practice, NORCE Norwegian Research Centre, Årstadveien 17, N-5009 Bergen, Norway; Department of Global Public Health and Primary Care, University of Bergen, Årstadveien 17, N-5009 Bergen, Norway; Division of Psychiatry, Haukeland, University Hospital, Haukelandsbakken 1, 5021 Bergen, Norway; Research Unit for General Practice, NORCE Norwegian Research Centre, Årstadveien 17, N-5009 Bergen, Norway; Department of Global Public Health and Primary Care, University of Bergen, Årstadveien 17, N-5009 Bergen, Norway

**Keywords:** depression, educational level, general practice, antidepressant drug treatment, large database research

## Abstract

**Background:**

Guidelines recommend follow-up within 2 weeks for patients starting medication for depression. Knowledge is lacking about how general practitioners’ (GPs) follow-up varies with patients’ sociodemographic characteristics.

**Objective:**

To describe follow-up by GP and specialist in mental healthcare provided to men and women with depression within 3 months of starting drug therapy. Furthermore, to examine whether follow-up varied according to patients’ age and education.

**Methods:**

Registry-based cohort study comprising all patients aged ≥18 years in Norway with a new depression episode in 2014 who started on antidepressants within 12 months from diagnosis. Patients’ age and educational level were the exposures. Outcomes were follow-up by GP and/or mental healthcare specialist, and talking therapy with GP, within 90 days of first prescription. Cox proportional hazard models were used to estimate the likelihood of having follow-up contacts. Log binomial regression analysis was performed to explore the likelihood of having talking therapy with a GP. Time to first contact was illustrated by Kaplan–Meier survival curves.

**Results:**

The study population comprised 17 000 patients, mean age 45.7 years, 60.6% women. Only 27.8% of the patients were followed up by GP and/or specialist within 2 weeks of the first drug dispensing, 67.1% within 90 days. Older or less educated men and women received less and later contacts than the younger or more highly educated.

**Conclusions:**

Differences in age and educational level were associated with follow-up of depressed patients who started medication. This may indicate unwarranted variation in depression care that GPs should consider when prescribing antidepressants.

**Key messages**

- Guidelines recommend follow-up within 2 weeks for depressed patients starting medication.
- Only one in four patients was followed up by a general practitioner or specialist within 2 weeks.
- Older patients had less contact and talking therapy than younger patients.
- Low-educated patients had less contacts and talking therapy than the highly educated.
- Clinicians should be aware of these differences to avoid unintended variations in depression care.

## Background

Depression is the most common reason for contact with general practice regarding mental health problems in Norway [1]. Treatment for depression usually consists of talking therapy and/or antidepressant medication. According to National Institute for Health and Care Excellence (NICE) guidelines, drug therapy should be considered for moderate or severe depression [2]. Follow-up after starting medication is recommended within 1–2 weeks by a general practitioner (GP) or mental healthcare specialist regarding compliance, treatment effects, side effects, and suicidality [2].

Studies from general practice in Australia and Denmark found that more patients with low income or low education received drug treatment for depression than patients with high income or high education [3, 4]. Registry-based studies in Norway showed that depressed women with high education were less likely to start drug treatment than women with low education, while no such differences appeared among men [5, 6]. Regarding age, more Norwegian patients younger than 30 years or older than 70 years received antidepressants than other age groups [6–8]. Furthermore, a study in the UK showed high antidepressant treatment rates among older patients with depression [9]. The variation in antidepressant drug treatment across sociodemographic patient groups does not have support in clinical guidelines [2].

There is little knowledge about whether this unwarranted variation also applies to follow-up after starting medication. One study from Denmark demonstrated that patients with low education or low income had less talking therapy with their GP and less contact with psychologists and outpatient psychiatrists within 12 months of starting antidepressant treatment [10]. As antidepressants are mostly prescribed by GPs [11, 12], follow-up in general practice is of particular interest. Filling the knowledge gaps can increase GPs’ awareness about variations in depression care and inform their decisions on how to improve follow-up.

This nationwide registry-based cohort study aimed to (i) describe GP consultation(s) with and without talking therapy and contact(s) with mental health care specialist given to men and women with depression within 3 months of starting drug therapy (follow-up), (ii) time to follow-up, and (iii) examine whether follow-up varied according to patient’s age and education.

## Methods

### Setting

Norway offers universal health care, ensuring all residents have equal access to medical care [13]. GP care is covered by the National Insurance Scheme. Patients 15 years+ must pay an out-of-pocket fee for consultations and reimbursed medication (e.g. antidepressant drugs), up to an annual maximum sum (219 € in 2014). GPs have fixed, personalized patient lists, and act as gatekeepers to specialist care.

### Study design

We conducted a nationwide registry-based cohort study that is part of ‘The Norwegian GP-DEP study’ [14]. The study cohort comprised all adult residents in Norway with a new depression diagnosis in general practice in 2014 who started drug treatment for depression within 12 months after diagnosis. The cohort was examined regarding GPs’ follow-up during the 90 days after patients’ first drug collection.

### Data sources

Information from five national registries for the study period lasting from 01 January 2011 until 31 December 2016 was linked at the individual patient level, using the (encrypted) unique personal identification number assigned to all residents in Norway. The data was pseudo-anonymized by a third party (Statistics Norway), meaning that enough personally identifiable information was removed or obscured so that the remaining information did not identify an individual. Data was stored and analysed on a safe server at the University of Bergen, accessible only to the researchers.

The study population was drawn from the *Population Registry*. We obtained information regarding gender and year of birth. Information on the highest level of completed education was retrieved from the *National Education Database*. From the *Control and Payment of Health Reimbursements Database (KUHR)*, we extracted information for each GP consultation, i.e. date of consultation, reimbursement code(s) for talking therapy, and all diagnoses according to the International Classification of Primary Care, 2nd version (ICPC-2) as recorded by the GPs [15]. The *Norwegian Patient Registry (NPR)* provided information on the date of all outpatient or inpatient contacts with mental health care specialists for depression, with diagnoses according to the International Classification of Disease, 10th version (ICD-10) [16]. The *Norwegian prescription database (NorPD)* stores information on all prescription drugs dispensed to patients treated in ambulatory care [17]. For each prescription of a depression drug, we obtained information on the date of dispensing, generic drug information (Anatomical Therapeutic Chemical code), and reimbursement code linked to specific diagnoses.

### Study population

The source population comprised the entire population of Norway born before 01 January 1996 and alive on 01 January 2008 (4 017 989 individuals) and was a closed cohort. First, we identified all individuals 18 years or older with a depression diagnosis recorded in a GP consultation (ICPC-2 code P76 Depression in KUHR) in 2014 (*n* = 128 565). Second, to establish a cohort of patients with a *new* depression diagnosis, washout was performed for patients with a depression diagnosis in general practice and/or specialist mental health care (ICD-10 codes F32, F33, F34, or F41.2 in NPR), and/or dispensed drugs reimbursed for the treatment of depression (antidepressants (ATC code N06A), selected antiepileptic drugs (N03A), and selected antipsychotic drugs (N05A) in NorPD) during 12-months *prior to date of depression diagnosis* (*n* = 53 225). Finally, among those with a new depression episode, we identified all patients who started drugs reimbursed for the treatment of depression within 12 months *from the date of depression diagnosis*, and who lived in Norway throughout the study period (*n* = 17 000).

### Independent variables

Patients’ age and educational level were the exposure. Age was categorized as 18–29, 30–39, 40–49, 50–59, 60–69, and ≥70 years. The National Education Database is based on the International Standard Classification of Education [18]. Eleven levels were recoded into three categories: low [primary school (grades 1–7) and lower secondary school (grades ≤8–10)]; medium (13 years, upper-secondary school); and high (>13 years, university, and higher education). Based on patients’ ICPC diagnostic codes recorded during 2011–2013, comorbidity was identified according to an established list of 38 common, chronic conditions, and categorized as none, 1–2, and 3 + conditions [19, 20]. This variable was used as a covariate/adjustment variable.

### Outcome

The observation period was days 1–90 after the first depression drug dispensing. We analysed follow-ups for depression by GP (consultations linked to ICPC code P76 in KUHR) and specialist in mental healthcare (inpatient and outpatient contacts linked to ICD-10 codes F32, F33, F34, or F41.2 in NPR). Among patients with GP consultation(s), we also analysed talking therapy by the GP (reimbursement code 615 for talking therapy linked to ICPC code P76 in KUHR). The term ‘talking therapy’ usually comprises various types of GPs’ psychological treatment, including supportive talk, counselling, and more structured psychotherapeutic methods such as cognitive-behavioural therapy [21–23]. In this paper, talking therapy is defined according to the reimbursement code 615 in the KUHR database as ‘talking therapy by a GP with a duration of at least 15 min with patients with mental disorders. The conversation must deviate from a normal conversation about medical issues and be of a therapeutic nature’ [24]. However, the reimbursement code 615 does not distinguish between different types of talking therapy’. Furthermore, we examined if the patients had GP consultations linked to ICPC codes other than P76 in the observation period. All outcome variables were binary (yes/no). Time from first drug dispensing to first contact for depression with GP (linked to ICPC code P76) and/or mental healthcare specialist (linked to ICD-10 codes F32, F33, F34, or F41.2) was counted in the number of days. Correspondingly, the time from the first drug dispensing to the first GP consultation for another diagnosis (linked to ICPC codes other than P76) was counted in the number of days.

### Statistical analysis

All analyses were performed for men and women separately.

Descriptive statistics was used to examine the distribution of age, education, comorbidity, and drug prescriptions, given by numbers and percentages of all patients, men, and women. The correlation between age and number of comorbid conditions was calculated using Pearson’s correlation coefficient.

Follow-up for depression with GP only, with mental healthcare specialist only, with GP *and* specialist, and with GP *and/or* specialist was provided by numbers and percentages, for age groups and educational levels. For patients without GP consultations for depression, consultation(s) with GP for diagnoses other than depression were given by percentages of all patients.

We used Cox proportional hazard models to estimate the likelihood of having follow-up with GP and/or specialist among the exposed. Reference groups were aged 18–29 years and low educational levels. Results are presented as hazard ratio (HR) with a 95% confidence interval (CI), crude and adjusted (adj) for age, education, and comorbidity.

Time to first follow-up contact with GP and/or specialist after first drug dispensing, was illustrated by Kaplan–Meier survival curves, for educational level and age, respectively. Individuals were censored if death (*n* = 65) occurred during days 1–90 after the first drug dispensing. Subanalysis was conducted stratified for patients who collected only one prescription, and for those who collected two or more prescriptions, respectively.

Among patients followed up by GP, consultations with and without talking therapy were provided by numbers and percentages, for age groups and educational levels. Log binomial regression analysis was performed to explore the likelihood of having talking therapy with a GP in follow-up consultations. Reference groups were aged 18–29 years and low educational levels. Results are presented as relative risk (RR) with 95% CI, adjusted for age, education, and comorbidity.

For all statistical analyses, *α* = 0.05 was used as the significance level. The data were analysed using STATA/SE version 18.0 (Stata Statistical Software).

## Results

The study population comprised 17 000 patients with a new depression diagnosis in general practice in 2014 who started antidepressants, 39.4% men [mean age 44.4 (standard deviation, SD 16.8) years] and 60.6% women [mean age 45.7 (SD 18.3) years] (Table 1). More women than men had completed high education (28.0% vs. 20.7%) and had one or more comorbid conditions (60.3% vs. 56.0%). There was a moderate positive correlation between age and comorbidity, *r* = 0.42. Of the study population, 71.2% collected two or more prescriptions within 1 year.

**Table 1.** Characteristics of patients with a new depression diagnosis and antidepressive treatment in general practice in Norway in 2014.

	All, *N* = 17 000		Men, *N* = 6696		Women, *N* = 10 304	
	*n*	%	*n*	%	*n*	%
Age, years						
18–29	4114	24.2	1609	24.0	2505	24.3
30–39	3251	19.1	1332	19.9	1919	18.6
40–49	3496	20.6	1445	21.6	2051	19.9
50–59	2637	15.5	1100	16.4	1537	14.9
60–69	1639	9.6	632	9.4	1007	9.8
70+	1863	11.0	578	8.6	1285	12.5
Educational level^1^						
Low	5539	33.1	2293	34.2	3246	31.5
Medium	6937	41.4	2919	43.6	4018	39.0
High	4268	25.5	1385	20.7	2883	28.0
* Missing*	256		99		157	
Comorbid conditions						
None	7045	41.4	2949	44.0	4086	39.7
1–2	8334	49.0	3154	47.1	5167	50.2
3+	1646	9.7	593	8.9	1051	10.2
Prescriptions^2^						
1	4899	28.7	1959	29.3	2940	28.5
2+	12 101	71.2	4737	70.7	7364	71.5

^1^Educational level: low (primary school [grades 1–7] and lower-secondary school [grades ≤ 8–10]); medium (13 years, upper-secondary school); and high (> 13 years, university, and higher education).

^2^Within one year from a new depression diagnosis in general practice.

In total, 67.6% of men and 66.8% of women received follow-up for depression by GP and/or mental healthcare specialist within 90 days (Table 2). The percentage decreased with older age and lower education.

**Table 2.** Follow-up with GP and mental healthcare specialist in Norway 2014–2016, during day 1–90 after first prescription of an antidepressant drug, numbers, and percentages given by age group and educational level (*N* = 16 953)^1^

		GP only		Mental healthcare specialist only		GP and mental health care specialist		GP and/or mental health care specialist^2^	
Men (*N* = 6667)	*n*	*n*	%	*n*	%	*n*	%	*n*	%
	6667	3742	56.1	259	3.9	528	7.9	4529	67.9
Age, years									
18–29	1609	847	52.6	103	6.4	152	9.5	1102	68.5
30–39	1331	749	56.3	47	3.5	124	9.3	920	69.1
40–49	1444	852	59.0	48	3.3	115	8.0	1015	70.3
50–59	1096	634	57.9	31	2.8	92	8.4	757	69.1
60–69	625	372	59.5	16	2.6	31	5.0	419	67.1
70+	562	288	51.5	14	2.5	14	2.5	316	56.2
Educational level^3^									
Low	2284	1245	54.5	87	3.8	164	7.2	1496	65.5
Medium	2907	1625	55.9	122	4.2	243	8.4	1990	68.5
High	1377	826	60.0	48	3.5	111	8.1	985	71.5
									
Women (*N* = 10 286)	*n*	*n*	%	*n*	%	*n*	%	*n*	%
	10 286	5710	55.5	361	3.5	812	7.9	6883	66.9
Age, years									
18–29	2504	1319	52.7	167	6.7	283	11.3	1769	70.7
30–39	1919	1095	57.1	64	3.3	184	9.6	1343	70.0
40–49	2051	1210	59.0	55	2.7	181	8.8	1446	70.5
50–59	1535	904	58.9	44	2.9	95	6.2	1043	68.0
60–69	1006	539	53.6	19	1.9	42	4.2	600	59.6
70+	1271	643	50.6	12	0.9	27	2.1	682	53.7
Educational level									
Low	3233	1685	52.2	118	3.7	224	6.9	2027	62.7
Medium	4014	2297	57.2	136	3.4	305	7.6	2738	68.2
High	2882	1654	57.4	104	3.6	268	9.3	2026	70.3

GP = General practitioner.

^1^Forty-seven patients excluded from further analyses due to death in the observation period.

^2^Follow-up consultation with GP only, mental healthcare specialist only, or GP and mental healthcare specialist.

^3^Educational level: low [primary school (grades 1–7) and lower-secondary school (grades ≤ 8–10)]; medium (13 years, upper-secondary school); and high (> 13 years, university, and higher education).

Among the study population, 32.9% had no follow-up for depression with a GP or specialist; 18.9% had GP consultation(s) recorded with other diagnoses than depression, i.e. 14.0% had neither GP consultations for depression nor other diagnoses.

Survival analysis showed that 27.8% of the patients were followed up within 14 days of first prescription, 50.9% within 30 days, and 67.1% within 90 days. Patients who collected only one prescription (*n* = 4899) received later follow-up for depression than patients who collected two or more prescriptions (*n* = 12 101): 21.7% vs. 30.3% within 14 days, 39.7% vs. 55.4% within 30 days, 54.9% vs. 72.1% within 90 days (data/figures not shown).

### Age

**Figure 1.**
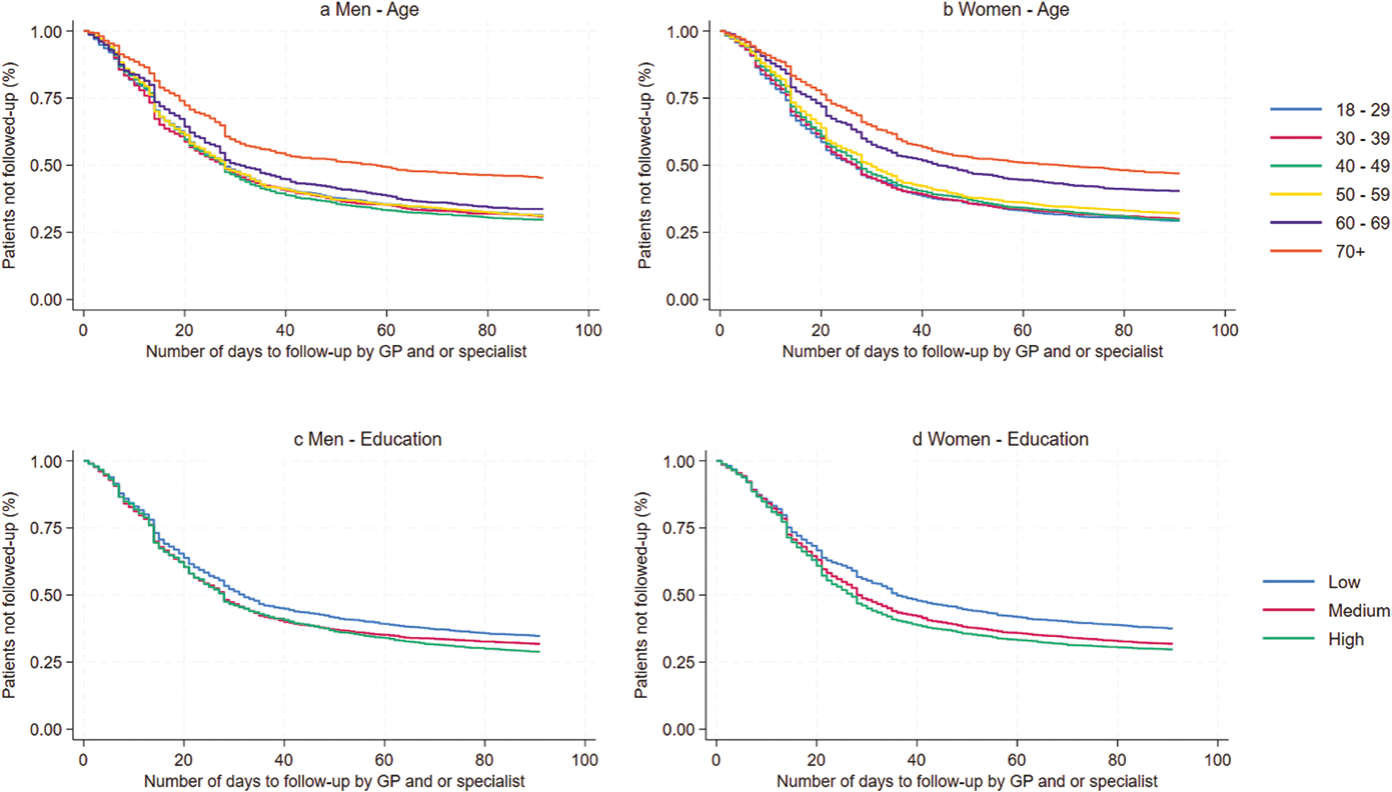
The association between age and educational level and number of days from first drug dispensing to follow-up consultation by GP and/or specialist (Kaplan–Meier survival curves) for patients in Norway, aged 18 years and older with a new depression diagnosis in 2014 (*N* = 16 953).

Men aged 70+ years and women aged 50+ years were less likely to have follow-up for depression compared to other age groups (Table 3). Survival curves showed that the contacts by age diverged immediately in the observation period. Among men aged 70+ years, 18.5% received follow-up by 14 days and 41.2% by 30 days, compared to 30.1% and 52.8% among men in all other age groups (Fig. 1a). Among women aged 60 years and older, 18.5% were followed up within 14 days and 38.5% within 30 days, compared to 29.3% and 53.7% among women in all other age groups (Fig. 1b).

**Table 3.** Likelihood^1^ of having follow-up with GP and/or mental healthcare specialist in Norway 2014–2016, during day 1–90 after first prescription of an antidepressant drug.

Men *N* = 6667			Follow-up with GP and/or mental healthcare specialist		Women *N* = 10 286		Follow-up with GP and/or mental healthcare specialist	
	*n*	%	Crude HR (95% CI)	Adj HRe (95% CI)^2^	n	%	Crude HR (95% CI)	Adj HRe (95% CI)^2^
Age groups								
18–29	1102	68.5	1	1	1769	70.6	1	1
30–39	920	69.1	1.03 (0.94–1.12)	1.02 (0.93–1.12)	1343	70.0	0.98 (0.91–1.05)	0.97 (0.90–1.04)
40–49	1015	70.2	1.04 (0.95–1.13)	1.04 (0.96–1.14)	1446	70.5	0.96 (0.90–1.03)	0.96 (0.90–1.03)
50–59	757	68.8	1.00 (0.95–1.10)	1.03 (0.94–1.14)	1043	67.9	**0.90 (0.83–0.97)**	**0.91 (0.84–0.99)**
60–69	419	66.3	0.92 (0.82–1.03)	0.96 (0.86–1.08)	600	59.6	**0.71 (0.65–0.78)**	**0.73 (0.67–0.81)**
70+	316	54.7	**0.68 (0.60–0.77)**	**0.74 (0.65–0.85)**	682	53.1	**0.59 (0.54–0.65)**	**0.64 (0.58–0.70)**
Educational level^3^								
Low	2027	65.2	1	1	2027	62.5	1	1
Medium	2738	68.2	**1.10 (1.03–1.18)**	**1.10 (1.03–1.18)**	2738	68.1	**1.17 (1.00–1.23)**	**1.16 (1.10–1.23)**
High	857	71.1	**1.15 (1.06–1.25)**	**1.13 (1.04–1.23)**	2026	70.3	**1.24 (1.17–1.32)**	**1.17 (1.10–1.25)**

Bolded data are statistically significant.

GP = General practitioner.

^1^Results from cox regression estimating HR with 95% CI.

^2^Estimates regarding age are adjusted for educational level and comorbidity; estimates regarding education are adjusted for age and comorbidity.

^3^Educational level: low (primary school [grades 1–7] and lower-secondary school [grades ≤ 8–10]); medium (13 years, upper-secondary school); and high (> 13 years, university, and higher education).

### Educational level

Men with medium and high education had a 10% and 13% greater likelihood of follow-up with a GP and/or specialist than those with low education, while women with medium and high education had a 16% and 17% higher likelihood compared to those low educated (Table 3). Survival curves showed that contacts by education diverged after 2 weeks. Among men with low education, 48.6% received follow-up within 30 days, compared to 53.9% of highly educated men (Fig. 1c). The corresponding figures for women were 44.7% of low educated, compared to 55.0% of highly educated (Fig. 1d).

### Talking therapy

Among the 10 792 patients who had GP consultation(s) for depression, 62.2% received talking therapy from the GP. The percentage decreased with older age and lower education. Men aged 60–69 years and 70+ years were less likely [RR = 0.83 (0.73–0.95) and RR = 0.69 (0.59–0.81), respectively] to have talking therapy, compared to the youngest men (Table 4). Men with high education were more likely [RR = 1.22 (1.13–1.22)] to have talking therapy than men with low education. Women aged 60–69 years and 70+ years were less likely [RR = 0.83 (0.75–0.91) and RR = 0.61 (0.54–0.68), respectively] to have talking therapy, compared to the youngest women. Women with medium and high education were more likely to have talking therapy, compared to women with low education [RR = 1.12 (1.05–1.19) and RR = 1.18 (1.11–1.26), respectively].

**Table 4.** Likelihood^1^ of having GP consultations with talking therapy in Norway 2014–2016, within 90 days of first prescription of antidepressant drug, among those who had follow-up consultation(s) with GP (*N* = 10 792).

	Men (*N* = 4270)					Women (*N* = 6522)				
	Talking therapy					Talking therapy				
	Yes		No		RR^1^	Yes		No		RR^1^
Age groups	***n***	**%**	***n***	**%**		***n***	**%**	***n***	**%**	
18–29	631	63.2	368	36.8	1	1045	65.2	557	34.8	1
30–39	599	68.6	274	31.4	**1.12 (1.03–1.22)**	835	65.3	444	34.7	1.02 (0.96–1.10)
40–49	629	65.1	338	35.0	**1.10 (1.01–1.20)**	898	64.6	493	35.5	1.03 (0.96–1.11)
50–59	434	59.8	292	40.2	1.01 (0.91–1.11)	642	64.3	357	35.7	1.01 (0.93–1.09)
60–69	203	50.4	200	49.6	**0.83 (0.73–0.95)**	342	58.9	239	41.1	**0.83 (0.75–0.91)**
70+	146	48.3	156	51.7	**0.69 (0.59–0.81)**	303	45.2	367	54.8	**0.61 (0.54–0.68)**
Educational level^2^										
Low	845	60.0	564	40.0	1	1135	59.5	774	40.5	**1**
Medium	1119	59.9	749	40.1	1.05 (0.98–1.12)	1603	61.6	999	38.4	**1.12 (1.05–1.19)**
High	642	68.5	295	31.5	**1.22 (1.13–1.32)**	1278	66.5	644	33.5	**1.18 (1.11–1.26)**

Bolded data are statistically significant.

GP = General practitioner.

^1^Adjusted results from binomial regression estimating RR with 95% CI, estimates regarding age are adjusted for education and comorbidity; estimates regarding education are adjusted for age and comorbidity.

^2^Educational level: low [primary school (grades 1–7) and lower-secondary school (grades ≤ 8–10)]; medium (13 years, upper-secondary school); and high (> 13 years, university, and higher education).

## Discussion

### Main findings

In a cohort of adult patients with a new depression diagnosis who started medication, we examined the association of age and education with GP consultation(s) and/or mental healthcare specialist contacts within 90 days of the first drug collection. In conflict with international guidelines, only 28% of the study population were followed up within 2 weeks [2]. Older or less educated men and women had later contacts than the younger or more educated. Among patients having GP consultations, the proportion who received talking therapy with the GP was higher among younger and higher educated patients.

### Strengths and limitations

The main strength of this study is the use of nationwide registry data, providing rich information and avoiding recall bias. We used depression diagnosis and reimbursement code for talking therapy as recorded by GPs, which reflect clinical practice. Although differing coding behaviour by GPs may challenge the internal validity, potential misclassification would probably be non-differential and distributed randomly across population groups. The KUHR database contains little clinical information apart from diagnoses and several diagnostic and therapeutic measures. Hence, we cannot know whether compliance, effect, and side effects of antidepressant medication or other topics have been raised by the patient or the physician in the follow-up consultation(s). Due to limitations in the Norwegian reimbursement coding system, we were unable to distinguish between different types of talking therapy provided by the GP. We lacked information on the severity of depression because grading is not available in ICPC-2. However, if drug treatment was mainly prescribed to patients with moderate to severe depression, we can assume some homogeneity in severity.

Education, income, and occupation are common measures of socioeconomic position. Level of education is an appropriate proxy because occupational social class and income to a large degree hinge upon education [25, 26]. As information was missing on income (9% of the study population and 42.1% in patients ≥60 years) and occupation (47% of the study population and 84% of patients ≥60 years), we used education as the only measure of socioeconomic characteristics.

From NorPD, we included drugs reimbursed only for the treatment of depression, and not for, e.g. anxiety disorders, to strengthen internal validity. NorPD contains data on dispensed medication, but we do not know whether patients used the drugs they collected. Because more prescriptions may indicate a greater likelihood of more severe depression, actual drug use, and more follow-up, we also estimated the time to follow up among patients who collected only one or several prescriptions, separately. The Norwegian GPs’ gatekeeper function probably leads to less treatment of patients with depression in specialist mental health care compared to other countries with direct access to secondary care. Consequently, our findings may be transferable, especially to countries with similar organizations of primary and secondary mental health care, such as other Nordic countries.

## Discussion of results

### Follow-up

As far as we know, this is the first study to investigate time to follow-up in general practice after starting medication for depression. Surprisingly, only 28% of the patients were followed up within 2 weeks, in conflict with international recommendations [2]. This is of concern because possible consequences may be (too) early self-discontinuation of medication, a more prolonged or more severe depression episode, and patients with existing or antidepressant-induced suicidal ideations going undetected [27, 28]. Possible reasons for no follow-up could be patients cancelling appointments due to remission or previous good experience with antidepressants, not starting or early discontinuing medication, using private specialist services (not covered by the registries), or inadequate follow-up by GP or specialist. The severity of depression probably affects the planning of follow-up, i.e. patients with more severe depression were seen earlier. The finding that patients who collected only one prescription received contacts later than those who collected more prescriptions points in the same direction. Although 67% of the study population had GP and/or specialist contact(s) for depression within 90 days, it is debatable whether late contacts can be considered as follow-up to the start of medication, unless the patient has recurrent depression and good experience of the medication working. Among the patients without contacts for depression, more than half had registered at least one consultation with a GP with another diagnosis. In these consultations, it is likely that the GPs have assessed the necessity of continued antidepressants or other treatment options, even if no depression diagnosis was recorded. GPs should inform patients about the importance of follow-up and facilitate new consultations for all.

### Variation in follow-up

Our finding that low education was associated with less contact with GP and/or specialist and less talking therapy by GP is consistent with a study from Denmark which found that patients with low education or low income received less talking therapy from their GP, and less contact with psychologist and outpatient psychiatrist [10]. To the best of our knowledge, there are no other studies on follow-up in general practice after initiation of drug treatment for depression. While Packness *et al*. [10] analysed contacts within 12 months of starting antidepressant drugs regardless of diagnosis, the present study examined contacts within three months of starting drugs for the treatment of depression diagnosed by a GP. Consistent findings across different countries and study designs lend support to the impact of educational level on depression care. Furthermore, our findings that older patients starting antidepressants received less consultations and talking therapy from GPs align with our previously published results about variation in depression care, regardless of whether the patients received medication [6–8]. Although our previous studies and the present study are based on the same registry data, the current study population starting on medication, represents a sociodemographic selection towards older age and lower education [3–8]. This fact supports the age and education gradient in GP depression care even more, indicating that some groups get more treatment and/or follow-up than others. The reasons for this treatment disparity remain unclear, but it suggests that inequity in depression care is maintained in patients starting medication, in conflict with international recommendations [2]. GPs should be aware that follow-up seems to favour younger and higher educated patients.

## Conclusions

Only one in four patients was followed up within 2 weeks of starting medication. Older and low-educated patients had less and later contact with GPs and/or specialists and less talking therapy with GPs than the younger and higher educated. Clinicians should be aware of these differences when prescribing antidepressant drugs and facilitate follow-up of all patients, to avoid unintended variation. We need further studies both from the GP perspective and from the patient perspective regarding expectations, the reality of daily GP practice, and perceptions from and about older and less educated patients about depression care. Clinical studies including information on the severity of depression and previous history of depression should explore the reasons for current variation in depression care and the impact on patient outcomes.

## Data Availability

The data underlying this article were provided by the Norwegian Directorate of Health, the Norwegian Institute of Public Health, and Statistics Norway by permission. The data cannot be shared publicly due to restrictions by the Norwegian Data Protection Authority.
